# Validity and reliability of the WHOQOL-BREF in the measurement of the quality of life of Sickle disease patients in Bahrain

**DOI:** 10.3389/fpsyg.2023.1219576

**Published:** 2023-09-01

**Authors:** Amer Almarabheh, Afif Ben Salah, Manal Alghamdi, Aseel Al Saleh, Abdulla Elbarbary, Ahmed Al Qashar, Faisal Alserdieh, Fatema Alahmed, Hasan Alhaddar, Lulwa Alsada, Mohamed Yosri, Mahmood Omran, Mina Khudhair, Motasem Salih, Noora Fuad, Sadok Chlif

**Affiliations:** ^1^Department of Family and Community Medicine, College of Medicine and Medical Sciences, Arabian Gulf University, Manama, Bahrain; ^2^Department of Medical Epidemiology, Pasteur Institute of Tunis, Tunis, Tunisia; ^3^College of Medicine and Medical Sciences, Arabian Gulf University, Manama, Bahrain

**Keywords:** WHOQOL-BREF, validity, reliability, quality of life, Sickle cell disease

## Abstract

**Background:**

Limited attention is devoted to the improvement of the quality of life of patients suffering from the negative consequences of Sickle cell disease (SCD). Our study focuses on the evaluation of the performance of the WHOQOL-BREF as a tool to measure the quality of life of SCD Patients in Bahrain.

**Methods:**

We conducted a cross-sectional study that enrolled 273 SCD patients selected using a simple random sampling technique from primary health-care centers in Bahrain in 2019. A designed questionnaire including the WHOQOL-BREF was filled by the patients in the health centers. The reliability of the WHOQOL-BREF was assessed by standardized Cronbach’s alpha coefficient, and the validity was measured by convergent validity, principal component analysis and confirmatory factor analysis.

**Results:**

The WHOQOL-BREF had good internal consistency as Cronbach’s alpha coefficient for the overall scale was 0.91. The convergent validity results indicated that the correlation coefficients values for all scale domains are significantly correlated at α < 0.01. Confirmatory factor analysis found that the four-domain structure produced a robust fit to the data.

**Conclusion:**

The WHOQOL-BREF tool has high internal consistency and validity in assessing the quality of life of Sickle Disease patients in Bahrain.

## 1. Introduction

Sickle cell disease (SCD) is an inherited autosomal recessive blood disorder that affects the structure of hemoglobin and is characterized by sickling of the red blood cells expressing abnormal hemoglobin-S due to genetic inheritance of homologous gene. This abnormal hemoglobin causes the red blood cells to assume a rigid sickle-shape in certain physiological and pathological conditions which in turn cause occlusion of blood vessels (Wailoo, 2017). It is a life-threatening condition that accounts for important morbidity load among children and adults worldwide (Brandow et al., 2017; Wailoo, 2017) and in Bahrain (El-Hazmi et al., 2011; Thein and Thein, 2016; Ali et al., 2017). SCD is responsible of high morbidity, mortality and demand of care in Bahrain where the prevalent cases more than 6,000 cases are registered in the electronic medical record system of the primary health care in 2023 according to the Primary Health Care Direction of the Kingdom of Bahrain. The vaso-occlusive crisis is responsible of sharp pain in different locations of the body. Disease related complications, recurrent pain crisis and repeated hospital admissions affected the mental health and the quality of life of SCD patients (AlSaleh et al., 2021).

The quality of life is defined as “individuals’ perceptions of their position in life in the context of the culture and value systems in which they live and in relation to their goals, expectations, standards and concerns” (The WHOQOL Group, 1998). The concept of quality of life (QoL) is widely used in a highly diverse range of disciplines and contexts (Merrill, 2015). The evaluation of Quality of life (Qol), as part of mental health, is nowadays considered as fundamental in all medical specialties and services (Barry and Crosby, 1996; Katschnig, 1997; Orley et al., 1998). The World Health Organization Quality of Life questionnaire (WHOQOL-BREF) is a tool developed to evaluate QoL in various areas of health care and different cultural settings, languages, and countries (Skevington et al., 2004; Saxena et al., 2005). It` is a 26 item self-report instrument derived from the original 100-item instrument, which had demonstrated reasonable validity and reliability. The WHOQOL-BREF evaluates four domains of quality of life (“Physical Health,” “Psychological Health,” “Social Relationships,” and “Environment”), and contains two other questions used to rate the individual’s “overall perception of quality of life” as well as his “overall perception of health” (Skevington et al., 2004). The reliability and validity of WHOQOL-BREF was assessed in several studies performed on subjects affected by depression (Berlim et al., 2005), alcoholism (Barros da Silva Lima et al., 2005), chronic psychiatric disorders (Herrman et al., 2002; de Willige et al., 2005), and psychiatric outpatients (Trompenaars et al., 2005). This tool, also showed satisfactory psychometric properties when used in heterogeneous samples (both in the general population and in those suffering from different diseases) (Power et al., 1999; Bulamu et al., 2015; Lodhi et al., 2017). Most of the validation studies revealed a four-factor structure of the WHOQOL-BREF scale (Vahedi, 2010). Similar to studies in Australia (Krägeloh et al., 2011) and China (Zhang et al., 2012), one recent study showed that the WHOQOL-BREF tool is valid (four domains) and reliable (the Cronbach’s alpha coefficient was over 0.7 for the questionnaire as a whole, and for all domains) for assessing quality of life among Saudi medical students (Malibary et al., 2019). The WHOQOL-BREF showed robust psychometric features in a recent study among patients having hip replacement surgery (Kumar et al., 2020). To our knowledge, only few studies addressed the performance of WHOQOL-BREF on SCD patients and this research has never been conducted on patients suffering from this condition in the gulf region including Bahrain where SCD is believed to account for a heavy psychological burden by family doctors. Therefore, we aimed in this study to assess the Validity and Reliability of the WHOQOL-BREF as a tool for the evaluation of the quality of life of life of Sickle Disease Patients in Bahrain. Thus, this user-friendly tool might become a recommended asset to monitor the QoL of these patients and respond timely to their needs.

## 2. Materials and methods

### 2.1. Study design and setting

A cross-sectional study conducted by our group and detailed elsewhere (AlSaleh et al., 2021) provided the required information for this study. Briefly, it enrolled (273 out 288 required according to the formula below) representative Sickle cell disease patients (above 21 years) from all primary health-care centers (PHC) in Bahrain between July and August 2019 who voluntarily accepted to fill the WHOQOL-BREF in the PHCs. The sample size was calculated using the following formula: *n* = (*Z*^2^ PQ)/*d*^2^ where *z* = 1.96, *p* = 0.25 and *Q* = 0.75, *d* = 0.05.

### 2.2. Study population sampling technique and data collection

The study population was described elsewhere (AlSaleh et al., 2021). The WHOQOL-BREF questionnaire was administered for eligible volunteers at the PHCs in Bahrain during the month of July and August 2019. They represent a sample selected using systematic random sampling from the database of SCD patients available in the PHC direction. The list of the patients available in the medical health records was used as a sampling frame from which the enrolled patients were selected using a random number list generated by computer. Patients were excluded if they have been diagnosed with any other health condition associated with chronic pain such as arthritis, chronic pain syndrome (CPS), low back pain (LBP), and/or psychiatric chronic diseases such as epilepsy, schizophrenia, and other mental illnesses. Vulnerable patients such as patients during the episode of pain crisis, pregnant women, or patients who refused or were unable to provide a valid informed consent were also excluded.

### 2.3. Study instruments

The Arabic version of WHOQOL-BREF questionnaire was used in this study to ensure good understanding by patients (Ohaeri and Awadalla, 2009). A total of (24) items: items 3–26 represent four domains: Physical Health (7 items), Psychological Health (6 items), Social Relationships (3 items), and Environment (8 items) are answered using a 1–5 Likert-type scale, where 1 denotes the least, and 5 is the highest agreement with a particular item (World Health Organization, 1996). Items 3, 4, and 26 are negatively phrased and reversed during analysis. The mean score of items within each domain is used to calculate the domain score. Results on domains represent the sum of results of items. A higher sum of points represents a higher quality of life on a single domain (Ilić, et al., 2019). Two other questions are asked separately and refer to an individual’s overall perception of quality of life and an individual’s overall perception of general health.

### 2.4. Statistical analysis

Convergent validity was assessed by the correlation between scores of the scales’ domains and perceptions of overall quality of life and overall general health using Pearson’s correlation coefficient. Construct validity was tested by exploratory factor analysis using principal components method relied on the extraction method, and the varimax rotation. The suitability of the data for this model was confirmed by the Kaiser-Mayer-Olkin (KMO) index and Bartlett’s sphericity test (BT) (Park, 2021). A factor was considered as important if its eigenvalue exceeded 1.0. Confirmatory Factor Analysis (CFA) using structural equation modeling was used to investigate construct validity. The goodness of fit was estimated by the χ^2^ test, the Root Mean Square Error of Approximation (RMSEA), the Comparative Fit Index (CFI) and Goodness of Fit Index (GFI). An RMSEA value <0.08 and a CFI value >0.90 indicated a good fit, also, for GFI a value >0.90 was an adequate model fit (Nia et al., 2016). Reliability analyses were carried out using Cronbach’s alpha coefficient to assess internal consistency reliability. All statistical analyses were conducted using the Statistical Package for the Social Sciences (SPSS) version 27, and the Analysis of Moment Structures (AMOS) software version 23. A value of *p* <0.05 was considered as statistically significant.

### 2.5. Ethics declarations

This study was approved by the Ethical Committee in the College of Medicine and Medical Sciences, Arabian Gulf University (approval number: E002-pi-4/19) and the ethical committee of the ministry of health in the kingdom of Bahrain (approval number: AURS/325/2019). All participants provided informed written consent before participation.

## 3. Results

### 3.1. Demographic characteristics of the study sample

The study sample was composed of 137 females (50.2%) and 136 males (49.8%). Regarding the education level, 157 (57.7%) of the respondents had secondary level of education or below, while 115 (42.3%) had BSc or high studies level of education. The mean age of the participants was 37.47 ± 10.47 years (range 20–70 years). The medical characteristics of SCD patients and quality of life level and determinants were detailed elsewhere (AlSaleh et al., 2021). The disease was mild among 54.6% of patients and did not require medication. However in 26.4 and 24.9% of cases non-steroidal anti-inflammatory drugs and opioids were prescribed, respectively, to relieve more severe pain crises.

### 3.2. Internal consistency reliability

The Reliability of the WHO quality of life as measured (Table 1) by Cronbach’s alpha coefficient (value = 0.91) was satisfactory considering all score items, and when considered separately, for “physical Health”, “psychological”, “social relationships”, and “environmental domains” (0.83, 0.72, 0.67, and 0.76 respectively).

**Table 1:** Reliability of WHOQOL-BREF overall and domains’ scores for SDC patients in Bahrain.

Domain	No. of items	Mean (SD)	Cronbach’s α coefficient
Physical Health	7	23.27 (5.33)	0.83
Psychological	6	22.56 (3.91)	0.72
Social relationships	3	11.97 (2.47)	0.67
Environmental	8	28.54 (5.31)	0.76
Overall	24	86.43 (14.62)	0.91

### 3.3. Convergent validity

Analysis indicated that the correlation between the different QoL domains is statistically significant and varied from 0.394 to 0.612 (value of *p* <0.001). Table 2 shows good convergent validity for the WHO quality of life domains as shown by the significant Pearson’s correlations coefficients between the WHOQOL-BREF domains and perceptions of “Overall Quality of Life,” and “Overall General Health”.

**Table 2:** Convergent validity of the WHO quality of life domains: correlation between scores of domains and overall quality of life perception of overall quality of life and overall general health.

WHO quality of life domains	Perception of QoL	Perception of overall general health
Physical health	0.384**	0.417**
Psychological	0.464**	0.492**
Social relationships	0.346**	0.268**
Environmental	0.424**	0.391**

**p < 0.01.

### 3.4. Construct validity

Results showed the Kaiser–Meyer–Olkin (KMO) Measure to be 0.882 and the Bartlett’s Test of Sphericity (BT) to be chi-square = 1744.66 (*p* < 0.001), which confirms the suitability of the data in this study for factor analysis (Table 3). The exploratory factor analysis using principal components method revealed four factors with eigenvalues over one explaining 52% of cumulative variance in the 24 items. The rotated solution shows that each factor accounted for 10.34% to 15.1% of the total variance. The factor loadings of the 24 items onto the four factors are shown in Table 3. Factor 1 included all the (7) items of the physical domain and 3 items: one item (negative feeling), and two items of the environment domain (Leisure activity, and Health care), and it explained 15.08% of the rotated variance. Four items of the original psychological domain, and 3 items: one item (Energy of life) of the original physical health domain, and two items (Security, and Leisure activity) of the original environment domain, were included in factor 2, and it explained 14.75% of the rotated variance. Factor 3 included all the (3) items of social relationships domain, and an item (satisfaction with self) of the original psychological domain, and it explained 11.83% of the rotated variance. Six items of the original environment domain, and an item (sexual activity) of the original social relationship’s domain, were included in factor 4, and it explained 10.34% of the rotated variance.

**Table 3:** Rotated factor matrix solution for factor analysis of 24 items.

Item number	Item description	Factor loading			
		1	2	3	4
	**Physical health**				
3	Pain and discomfort	**0.782**	0.108	−0.093	0.236
4	Need for medical treatment	**0.720**	0.099	−0.111	0.141
10	Energy for life	**0.362**	**0.648**	0.174	0.027
15	Mobility	**0.660**	0.378	0.150	0.120
16	Sleep and rest	**0.583**	0.272	0.443	−0.040
17	Activities of daily living	**0.555**	0.449	0.446	0.023
18	Work capacity	**0.516**	0.393	0.299	0.190
	**Psychological**				
5	Positive feeling	0.262	**0.643**	0.021	0.298
6	Personal belief	0.138	**0.633**	0.068	0.099
7	Concentration	0.087	**0.613**	0.128	0.094
11	Body image	0.159	**0.555**	0.257	0.098
19	Satisfaction with self	0.232	0.272	**0.524**	0.381
26	Negative feelings	**0.459**	0.133	0.043	0.278
	**Social relationships**				
20	Personal relationships	0.085	0.159	**0.752**	0.289
21	Sexual activity	0.306	0.042	**0.362**	**0.469**
22	Social support	−0.149	0.116	**0.777**	0.176
	**Environment**				
8	Security	−0.010	**0.662**	−0.045	**0.364**
9	Physical environment	−0.001	0.330	0.068	**0.651**
12	Financial support	0.153	0.268	0.167	**0.605**
13	Accessibility of information	0.286	0.250	0.149	**0.366**
14	Leisure activity	**0.362**	**0.503**	0.240	0.158
23	Home environment	0.165	0.149	0.146	**0.646**
24	Health care	**0.340**	0.077	0.288	0.250
25	Transport	0.212	−0.033	0.201	**0.688**
	**Eigenvalues**	7.94	1.82	1.44	1.29
	**Variance explained** (%)	15.08	14.75	11.83	10.34
	**Cumulative variance** (%)	15.08	29.83	41.66	52.00

All bold values >0.30 (0.30 was considered the least significant factor loading of the item according to Guilford Scale).

**Figure 1:**
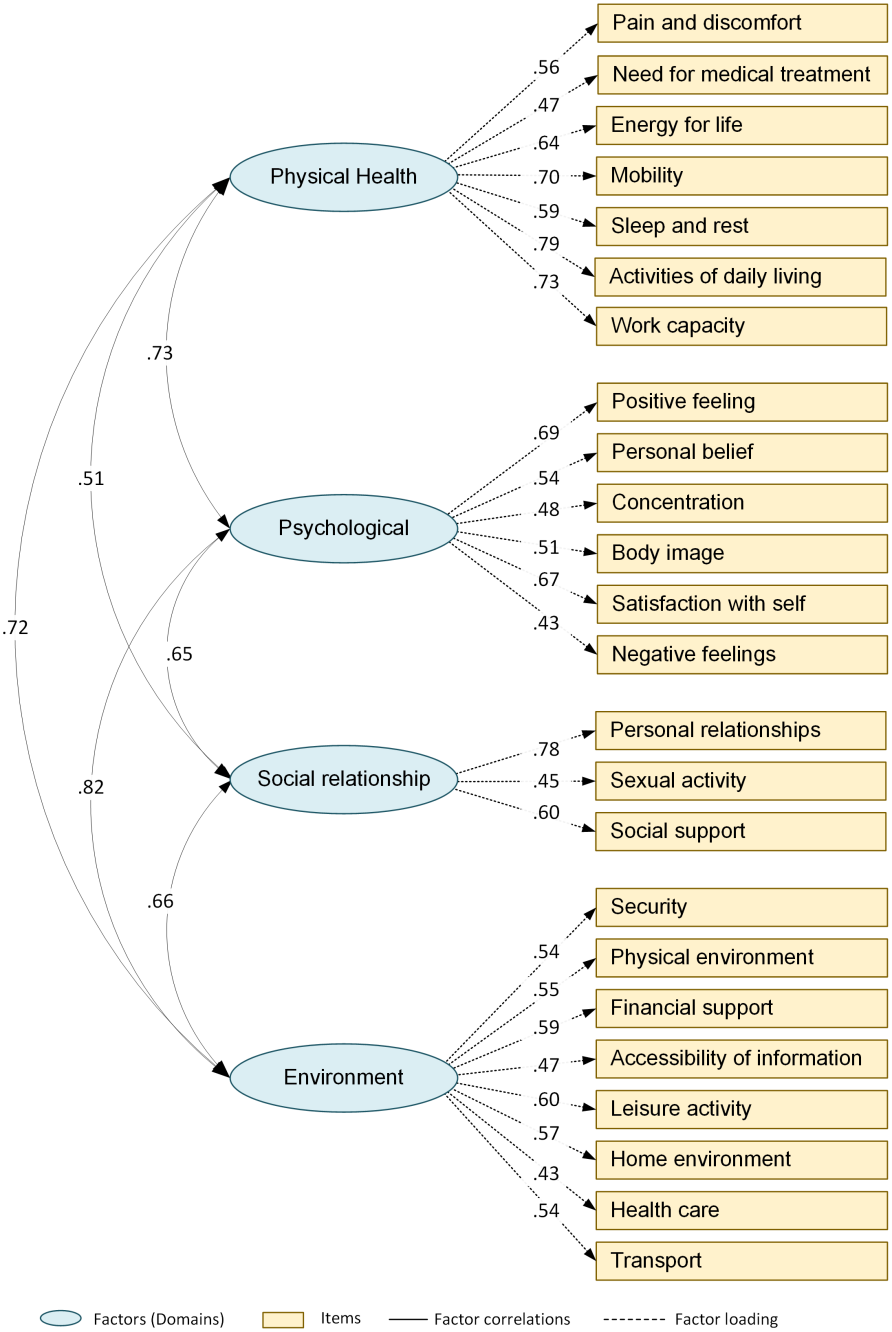
The structure of the WHOQOL-BREF based on confirmatory factor analysis.

The Confirmatory Factor Analysis (CFA) results showed that the four-domain structure of the WHOQOL-BREF produced a good fit to the data [χ^2^ = 343.707, df = 232 (χ^2^/df = 1.481, *p* < 0.001); CFI = 0.944; RMSEA = 0.042 and GFI = 0.907]. The factor load of each item with its respective domain ranged from 0.43 to 0.79 (Table 4). These values were adequate, and the observed model showed good fit with the theoretical one revealing a good construct validity of the tool (Figure 1), which provided statistically significant model.

**Table 4:** Fitness statistics for the four factor-analytic models of the WHOQOL questionnaire.

Model	χ^2^	df	χ^2^/df	GFI	CFI	RMSEA (90% C.I)
Original model	581.203	246	2.363	0.850	0.830	0.07 (0.04–0.08)
Modified model	343.707	232	1.481	0.907	0.944	0.042 (0.01–0.05)

χ2: chi-square; df, degree of freedom; CFI, comparative fit index; GFI, goodness of fit index; RMSEA, root mean square error of approximation.

## 4. Discussion

The aim of this study was to examine the validity and reliability of the WHOQOL-BREF in the evaluation of the quality of life of SCD using a representative sample of 273 patients suffering from this disease in Bahrain. The results of our study confirmed fair validity and reliability of the WHOQOL – BREF as a tool for the measurement of the quality of life of SCD patients in Bahrain. Indeed, Cronbach’s alpha coefficients for all domains (physical health, psychological, social relationships, and environmental) were 0.83, 0.72, 0.67, and 0.76, respectively. Except for the social relationship’s domain, the coefficients of the three domains were above acceptable value 0.7, which confirmed the good internal consistency of this tool (Privitera, 2017). A lower alpha coefficient of the social relationship’s domain ranging from 0.58 and 0.66 was also reported in other studies (Katschnig, 1997; Chung et al., 2012; Zhang et al., 2012). Similar findings were reported in multiple quality of life studies in Pakistan (Lodhi et al., 2017), Norwegian (Kalfoss et al., 2021), Saudi Arabia (Malibary et al., 2019), and Portugal (Goes et al., 2021). Lower alpha values for the social domain are most probably due to the unrelated and limited number of three items in this domain as proposed by Wilcox (2017), and more recently in India (Kumar et al., 2020).

Regarding convergent validity, our findings indicated that all values of the correlation coefficients were statistically significant. Also, the current study confirmed that the psychological domain has highly contributed for both overall quality of life, and perception of overall general health. Noticeably, in a study of patients with physical impairments in Korea, the psychological domain was the main contributor of overall quality of life, whereas the physical domain is highly associated with general health (Kim et al., 2013). A study of Polish respondents has also shown that the psychological domain has the strongest contribution for overall quality of life, whereas the physical domain was the contributor of general health followed by the psychological domain (Jaracz et al., 2006). However, the physical domain was found to be a strong contributor for both overall quality of life and general health in a study conducted in India among people with type 2 diabetes (Sreedevi et al., 2016). These different patterns could be explained by the nature and specificities of the related health problems such as patients on wheelchairs with heavy handicaps caused by muscular skeletal dystrophies or elderly with hemiplegia.

Regarding the validity, the results of exploratory factor analysis (EFA) showed the presence of four main factors with an eigen value greater than 1, explaining 52% of variance. These findings corroborate in the results of the international study (WHOQOL-BREF field trial), where the four factors explained 53% of variance (Skevington et al., 2004). However, they are slightly lower than those reported from a study conducted in China, where the four domains cumulative contribution was 69.3% (Zhang et al., 2012).

The confirmatory factor analysis (CFA) provided an acceptable fit to a four-factor model in the sickle disease patient’s sample. Regarding the CFA, the original model has indicated that the domains in model did not fit for the sickle disease patients. However, the model gained acceptable goodness of fit after three pairs of error variance were allowed to covary, i.e., item 19 (psychological) and item 20 (Social relationship); item 5 (psychological) and item 14 (Environment); item 16 (physical health) and item 24 (Environment); and after three pairs of items were allowed to cross-load on other domains: physical health domain and item 14 (Environment); social relationship domain and item 19 (psychological); psychological domain and item 8 (social relationship). This pattern is in line with the findings of different studies which used WHOQOL-BREF when pairs of error variances could covary and some items were allowed to cross-load on other domains (Hwang et al., 2003; Fu et al., 2013; Reba et al., 2019).

### 4.1. Strengths and limitations

This is the first study to measure the psychometric properties of the WHOQOL-BREF for SCD patients in Bahrain, and this area has never been addressed previously although SCD is highly prevalent. The study was limited by the retrospective character of the cross-sectional design which might lead to a recall bias. Young patients and the most severe ones were not included in the present study for ethical and feasibility reasons which might compromise the generalizability of findings. Future prospective studies using larger samples and enrolling patients from secondary care are required to have a more valid and representative evaluation of the tool.

## 5. Conclusion

This study confirmed the usefulness of the WHOQOL-BREF in the measurement of the quality of life of SCD patients using a representative sample from Bahrain. It revealed good psychometric properties (Reliability & Validity) and should be recommended for future use in this health condition impact assessment on patients. However, more studies are required to improve the reliability results particularly in the “social relationship” domain where the number of items is relatively reduced. This gap might be addressed using a qualitative study design approach which is favored because of its inductive nature in generating items within constructs. Quantitative prospective studies on larger samples of patients including the whole severity spectrum of SCD are required. Despite these limitations, we recommend the integration of WHOQOL-BREF in the practice as a rapid tool to evaluate the quality of life for SCD patients at the point of care and timely address their needs.

## Data availability statement

The original contributions presented in the study are included in the article/supplementary material, further inquiries can be directed to the corresponding author.

## Ethics statement

This study was approved by the Ethical Committee in the College of Medicine and Medical Sciences, Arabian Gulf University (approval number: E002-pi-4/19) and the ethical committee of the ministry of health in the kingdom of Bahrain (approval number: AURS/325/2019). All participants provided informed written consent before participation.

## Author contributions

AA and ABS conceived and designed the study. AE, AQ, FtA, FiA, MS, and MO performed the research process and collected the data. AA performed the statistical analyses. AA, ABS, SC, and MA wrote the original draft of the manuscript. AA, ABS, and SC prepared the figures and tables. AS, HA, LA, NF, MY, and AA edited and revised the manuscript. AA and ABS was the project manager. All authors contributed to the article and approved the submitted version.

## Conflict of interest

The authors declare that the research was conducted in the absence of any commercial or financial relationships that could be construed as a potential conflict of interest.

## Publisher’s note

All claims expressed in this article are solely those of the authors and do not necessarily represent those of their affiliated organizations, or those of the publisher, the editors and the reviewers. Any product that may be evaluated in this article, or claim that may be made by its manufacturer, is not guaranteed or endorsed by the publisher.

## Figures and Tables

**Table:** 

QoL	quality of life
SCD	Sickle cell disease
PHCs	primary health-care centers
CPS	chronic pain syndrome
LBP	low back pain
EFA	exploratory factor analysis
CFA	confirmatory factor analysis
KMO	Kaiser-Mayer-Olkin
CFI	comparative fit index
GFI	goodness of fit index
RMSEA	root mean square error of approximation
BT	Bartlett’s sphericity test
AGU	Arabian gulf university
CMMS	college of medicine and medical sciences
χ2	chi-square
df	degree of freedom
